# A review of software for analyzing molecular sequences

**DOI:** 10.1186/1756-0500-7-830

**Published:** 2014-11-24

**Authors:** Haema Nilakanta, Kimberly L Drews, Suzanne Firrell, Mary A Foulkes, Kathleen A Jablonski

**Affiliations:** The Biostatistics Center, The George Washington University, 6110 Executive Boulevard 750, Rockville, MD 20852-3943 USA; Department of Epidemiology and Biostatistics, The Milken Institute School of Public Health, The George Washington University, Washington, DC USA

**Keywords:** Microbiome, Molecular sequencing, Analytic pipelines, 16S gene

## Abstract

**Background:**

Over the past ten years, there has been an explosion of microbiome research. Many software packages for analyzing microbial sequences such as the 16S gene from 454 sequencers and Illumina platforms are available. But for a new researcher, it is difficult to know which package to choose. We present a systematic review of packages for the analysis of molecular sequences used to describe and compare microbial communities. This review gives students and researchers information to help choose the best analytic pipeline for their project. To the best of our knowledge, this is the first review of such software.

**Findings:**

Seven software packages met our inclusion criteria of being cost free and publically available, offering analysis functions from platform sequencing to results presentation, and included documentation and data security. We installed and executed each of the software packages and describe the installation, documentation, features, and functions of each.

**Conclusions:**

For the user, pipeline choices may be limited because some packages only run on select operating systems. Users should be aware of the availability of features and functions of each package. Of utmost importance is that the user must be aware of the default settings and underlying assumptions of each function. All packages are lacking sufficient methods for longitudinal analysis.

Researchers can do well using any one of these seven packages. However, two packages are outstanding; mothur and QIIME, due not only to the comprehensive suite of functions and procedures incorporated into the pipelines but also because of the accompanying documentation.

## Findings

With the proliferation of microbiome research, software packages for analyzing sequences such as the 16S gene from 454 sequencers and Illumina platforms have become available. We present a systematic review of packages for the analysis of metagenomic sequencing used to describe and compare microbial communities. Pipelines are programs that wrap around and call other programs so a user is freed from the trouble of downloading and installing each program separately. The purpose of this review is to give students and researchers information to help them choose the best analytic pipeline to use for their project and provide them with a roadmap to make future choices beyond the pipelines discussed here. This paper aims to catalog features and functions of pipelines but does not address performance. Because of the availability of multiple processors on computers, even laptops, performance is not an important consideration. We also believe that pipeline choice should be based on the available features and functions and not speed. To the best of our knowledge, this is the first review of such software.

### Pipelines

Seven analytic pipelines met all four of our inclusion criteria: mothur, QIIME, WATERS, RDPipeline, VAMPS, Genboree, and SnoWMan. The web addresses for each package are given below. Of these, mothur [1] and QIIME [2] are the most well known sequencing analysis packages [3, 4]. The authors of mothur rewrote specific program tools and algorithms to optimize software included in the package. In contrast, the creators of QIIME combined original published tools and algorithms directly into the pipeline [5]. WATERS [6] was developed with the main objective to expand sequencing analysis to non-bioinformaticians. RDPipeline [7] was created for high-volume amplicon sequencing data. VAMPS [4] was also designed to allow ecologists and clinicians to easily analyze sequencing data with a "point and click" interface. Genboree [8] likewise was created for individuals of all bioinformatic levels. Lastly, SnoWMan [9] was designed with a straightforward analysis interface and basically operates as a pipeline of pipelines. We installed, followed tutorials, and assessed the documentation for each pipeline. Details of their functions and differences are found below.

### Installation

System requirements for installation differ among the pipelines. While some are built and maintained for specific operating systems, others can operate on multiple systems. For example, mothur and QIIME have native versions that users can install on Mac OS X, Windows or Linux operating systems. As the installation process can be a bit involved, both of these pipelines also have "direct download" versions allowing the user to simply download one main file to install the program. For mothur this is an executable file, and for QIIME this is a VirtualBox, both of which work with multiple systems.

WATERS was built and tested for Mac OS X 10.5 and 10.6 only. The developers state that you can download it to other systems, but that it may not work properly. RDPipeline has both a web-based version and the option for the user to download the tools used in the RDPipline to make their own workflow. For this review, we will focus on the web version of the RDPipeline. The remaining pipelines featured (VAMPS, Genboree and SnoWMaN) are fully web-based, and require no installation process, other than a reliable working internet connection and strong bandwidth.

All of the pipelines discussed have online guides and/or wiki pages that provide step by step installation instructions and/or FAQs for the user. Additionally, several of the pipeline websites also host a tutorial page that have a trial sequencing dataset that users can download to test and practice executing pipeline functions.

### Updating

Users need to be informed of updates to a pipeline as new version releases may resolve programming bugs, improve existing applications and/or introduce new functions. Announcements for new releases are usually posted on a pipelines’ website homepage, but each package may have different updating procedures. For instance, if the user decides to base install QIIME, thereafter it is the responsibility of the user to regularly check for new releases of any applications, and update them as needed. To update the direct download versions of both mothur and QIIME, the software must be reinstalled by downloading the software again. WATERS appears to update in a similar manner but there have been no recent updates to the software.

The developers of web-based pipelines update individual applications as they become available. Users of mothur and QIIME, however, must download new versions of the pipeline as they become available. Users should be aware of which version of a program the pipeline has integrated into its system at any time. Differing results from identical runs of a pipeline may be caused by executing different versions of the same program.

### Features

As stated earlier, the purpose of analytic sequencing packages is to streamline the process of sequencing curation, filtering, analysis and output. There are several capabilities that are integral to this process. In Table 1 we present some of the major capabilities across the seven pipelines reviewed. We left cells empty if they did not apply to the pipeline or there was no documentation for that specific feature. This table serves as a resource to help researchers decide which pipeline to use when starting their own projects. The web addresses for each pipeline are given at the end of this article.

**Table 1:** **Major functions of seven pipelines**

Capabilities		Mothur	Qiime	Waters	RD-Pipline	VAMPS ^‡^	Genboree ^‡^	SnoWMAn ^‡^
Documentation	Available guides	✓	✓	✓	✓	✓	✓	✓
Installation	Shortcut option (instant download)	✓	✓					
	Native version: Mac OSX	✓	✓	✓^**†**^				
	Native version: Windows	✓	✓	✓				
	Native version: Linux	✓	✓	✓				
	Web based				✓	✓	✓	✓
Updating	Re-download entire program	✓	✓	✓				
	Re-download updated sections		✓					
Interface	Command line	✓	✓					
	Graphical User Interface			✓				
	Web form GUI				✓	✓	✓	✓
Sequencing platforms	Illumina	✓	✓	✓	✓^**†**^	✓	✓	✓
	454 Pyroseq	✓	✓	✓	✓	✓	✓	✓^**†**^
Preparing sequences	Accepted file formats							
	sff	✓*	✓*		✓	✓	✓	
	Fasta	✓	✓	✓	✓	✓	✓	✓
	Quality score	✓	✓		✓			✓
	Flow file data	✓	✓					
	User defined barcodes or primers	✓	✓	✓	✓	✓	✓	✓
	User defined metadata	✓	✓	✓	✓	✓	✓	✓
	Alignment	✓	✓	✓	✓	✓	✓	✓
	Summary function	✓	✓		✓			
	Trims barcodes and primers off of sequences	✓	✓	✓	✓	✓	✓	✓
	Removes short reads	✓	✓	✓	✓	✓	✓	✓
	Identify and remove chimeras	✓	✓	✓	✓	✓	✓	✓
	Remove contaminants	✓	✓					
Approaches to analyze files	OTU binning/clustering	✓	✓	✓	✓	✓	✓	✓
	Phylotype binning	✓	✓					✓
	Phylogenetic tree	✓	✓	✓			✓	
Analysis output	Alpha diversity	✓	✓	✓	✓	✓	✓	✓
	Beta diversity	✓	✓	✓	✓	✓	✓	✓
	Ecological indexes	✓	✓	✓	✓	✓	✓	✓
	Unifrac	✓	✓	✓	✓	✓	✓	✓
	Visualization	✓	✓	✓	✓	✓	✓	✓

*Not required but strongly suggested; ^†^Engineered for specific capability; ^‡^Integrates other pipelines

Three pipelines, VAMPS, Genboree and SnoWMan have other pipelines built into them. VAMPS integrates QIIME and mothur, Genboree has both QIIME and RDPipeline, meanwhile SnoWMAN integrates mothur, RDPipeline and other pipelines not featured in this review.

### Specific capabilities

#### Quality Score File

A first step in filtering sequences is to trim sequences with low quality scores. A Quality score is a product of the Phred program, which is now included in most Next Generation sequencing (NGS) platforms. Phred [10] assigns a score that indicates the probability of an incorrect base call to each base of a sequence. The higher the quality score, the lower probability of an incorrect base, and hence the greater base call accuracy. The Solexa quality score (for early Illumina) is calculated in a similar manner to Phred, although is no longer used much [11].

After sequencing, the platform creates a file that contains the quality scores. For Roche-454 platforms this is the QUAL file and for the Illumina platform this is the FASTQ file. Not all NGS platforms use the same quality score scales though. For example, Roche-454 QUAL files use the score from the Phred program, but the Illumina FASTQ quality scores offset the Phred scores by 64 to follow ASCII characters [11].

Currently, there is no standard cut off point for quality score. Default cutoff limits vary between pipelines (see Table 2). The ideal cutoff is something the researcher needs to choose and consider how it will filter sequences [12].

**Table 2:** **Defaults for each of the reviewed pipelines**

	Mothur	QIIME	WATERS	RDPipeline	VAMPS	Genboree	SnoWMAn
**Minimum reads**	0 – User defined	200 bp	500 bp	150 bp	200 bp	0 – User defined	150 bp
**Maximum reads**	Not bounded – User defined	1000 bp		500 bp	1000 bp	Not bounded – User defined	
**Max homopolymers**	Not bounded – User defined	6 bp					
**Number of allowable ambiguous bases (AB) or N’s**	Not bounded - User defined (recommended AB = 0)	AB = 6		N = 0	N = 0	Not bounded – User defined	N = 0
**Minimum quality score**	0 – User defined	25		20		0 – User defined	20
**Alignment method**	Needleman	Pynast	Infernal	Infernal	GAST	Pynast	Varies by pipeline method
**Chimera**	User defined	User defined	Mallard	Uchime		ChimeraSlayer	

bp = base pairs.

#### Chimeras

Another capability of great interest is a pipeline’s ability to remove chimeric sequences (or chimeras for short). Chimeras occur during the DNA PCR amplification process when two or more different parent DNA strands combine. This creates a new hybrid sequence and results in false diversity in the final sequencing set [13]. Several programs feature different methods to remove chimeras from sequencing data sets. Edgar et al. [14], reference many of the chimera finding programs in their paper introducing the UCHIME program, but we have added additional programs to their list (see Table 3). One of the original programs, CHIMERA_CHECK [15] was designed with the Ribosomal Database Project (RDP) to identify chimeras. Another initial program was Bellerophon [16] which identified chimeras using a partial treeing approach, where branches are compared for incongruencies that may highlight chimeric sequences.

**Table 3:** **Summary of programs to remove chimeras**

Chimera program	Year published	Method	Advantage
CHIMERA_CHECK [15]	1999		Initial program
Bellerphon [16]	2004	Partial treeing approach	Initial program
Pintail [17]	2005	Reference database comparing variation differences	More sensitive than earlier methods
Ccode [19]	2005	Reference of putative chimeras, measuring variability	Bypasses need for manual inspection
Mallard [18]	2006	Reference database comparing variation differences to all pairs	More sensitive than earlier Pintail program
ChimeraChecker [20]	2010	Focuses on ITS region using BLAST	Used for fungal sequences
ChimeraSlayer [12, 21]	2011	Reference database constructs potential alignments with parent strands	Useful for short sequences and where parents of chimeras are closely related – more sensitive than earlier methods
Perseus [12, 22]	2011	Searches for parts of parent sequences in higher abundance	*de novo* sequences from 454 pyrosequencing reads
UCHIME [12, 14]	2011	Uses multiple reference databases, aligning to top hits and computes score	Faster without sacrificing sensitivity, identifying chimeras with more than two parents
DECIPHER [23]	2012	Search-based approach, detecting short fragments	Useful for short sequences

Currently, newer programs are becoming more popular for chimera detection. Both the Pintail [17] and Mallard [18] programs use a reference database that contain chimera-free reference sequences. Query sequences are aligned with the chimera-free reference sequences. Using a sliding window, variations in evolutionary distance are computed with the known rate of variability in the 16S gene, such that larger variations indicate a chimeric sequence. Ccode [19] also uses a reference database. It takes putative chimeric sequences with relation to their closest reference sequences in the database; where the variability between the references is compared to the variability between the query sequence and the references. Another method used by ChimeraChecker [20] looks at the fungal internal transcribed spacer (ITS) region of the sequence and compares it to a reference database to identify chimeras. In contrast, ChimeraSlayer [21] created with Sanger and 454 pyrosequencing platforms in mind, goes through several alignments of chimera-free reference sequences and then constructs alignments with potential parent strands. Meanwhile, Perseus [22] works with *de novo* 454 sequences that have been filtered by the AmpliconNoise program, to identify the parent sequences that were combined into the chimeras since they will be in higher abundance in the sequencing dataset. UCHIME [14] uses multiple reference databases, aligns the query sequence to the top two reference hits, and if the query alignment is greater than a certain percentage, a score is computed and if that score crosses a certain threshold, the query sequence is marked as a chimera. This program can also work with *de novo* sequences, using a system similar to Perseus. Lastly, DECIPHER [23] uses a search-based approach. It works by identifying short fragments that are uncommon in the phylogenetic group where the query sequence is from, but that are found in other phylogenetic groups.

Some sequencing pipelines have a default chimera program (refer to Table 2). If not, users must specify what program they would like to use. Also, certain programs work better with short sequences versus long and vice versa. It is very important that the user understands both the advantages and disadvantages of their selected chimera program and how that will affect their downstream analysis.

#### Removing contaminants

Another step to filtering datasets is removing contaminants, i.e. outside sources of microbes not native to the sample. A common practice in many studies is to sequence control samples from the source environment. These OTUs are then removed from the analysis dataset. In other words, if an OTU is present in both the analysis and control sets, it can be discarded from the analysis set as coming from an outside source [1]. Another contaminant finding method is to use the program SourceTracker that was first tested with QIIME and is available as an R package [24]. SourceTracker takes a Bayesian approach to estimate the proportion of contaminants in an analysis set given the source community [24]. Contaminants can overestimate microbe diversity in a sample if not accounted for in the filtering process, therefore it is important for the researcher to adjust for contamination when preparing OTUs for analysis.

### Important defaults

A common theme that emerged while reviewing each pipeline is the importance of default settings, especially in the quality filtering process. Quality filtering reduces researchers’ sequencing datasets down to smaller sets that are used in final analyses and shape the results for publications and future work. The parameters used for filtering may affect final analysis sets which in turn may affect the end results. Hence, researchers should be aware of these default settings in the filtering process. This may also play a role in reproducing results, and thus is important information to include in any publication of results. In Table 2 we present the documented defaults of the seven pipelines. If we could not find a default setting, or any documentation, an empty cell is shown.

The authors of software packages could improve documentation by offering dedicated web pages for default functions and their default parameters. This would also help in standardizing sequencing analysis.

### Analysis options

All the pipelines we reviewed offer the usual array of analyses typically used in ecological studies such as ecological indices including alpha and beta diversity measures [25, 26] and tools for comparing phylogenetic information such as unifrac [27]. The pipelines provide adequate methods for cross-sectional studies, however, none of them contain methods for analyzing studies that are repeated measures (i.e. multiple samples from the same subject and longitudinal studies) designs. Users need to be cautious about reporting results from statistical tests that do not adjust the standard errors for such correlations as standard errors will typically be smaller resulting in an inflated type I error rate [28]. No pipeline offers mixed model analysis which is useful in analyzing longitudinal studies with missing data.

## Discussion

Although we had expected to find a limited number of pipelines that met our inclusion criteria, we identified seven programs for analyzing 16S rRNA sequences with the desired characteristics. All of these software packages are available for free and are able to analyze data from platform to results. Many more programs are available that did not satisfy our inclusion criteria.

To guide the user in the selection of a package, we have compiled a listing of the capabilities of each program (see Table 1) so it is easy to compare and contrast features. This table provides guidelines for future pipelines that were not available to include in this comparison. We think that a user should not choose software only because it is was used in the lab on a previous project, but should consider the capabilities that are present for the current project.

**Figure 1:**
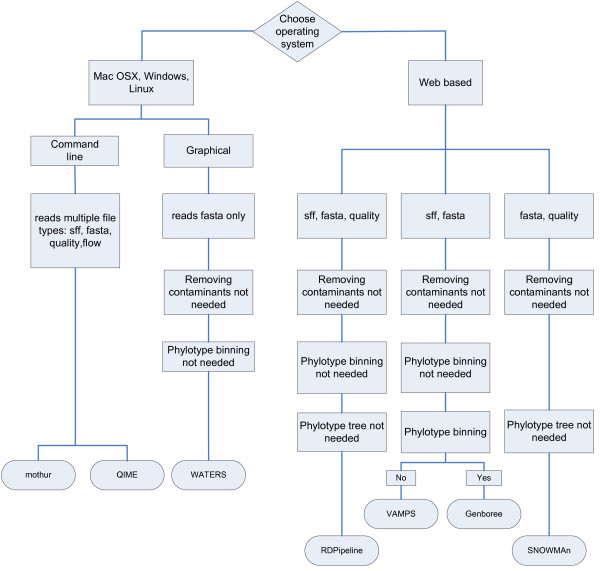
**Decision tree showing feature options for pipeline choice (based on documented features within each package).**

Practical issues will limit the choice of which package to use as not all packages work on all operating systems. The installations procedures for some of the software may be too complicated for users without a lot of computer training so a user will do well to choose a package with short cut options or one that runs on the web. Software selection may also be limited by the type of file formats that a program can accept. Serious consideration should be given to the available functions and categorization of microborganisms (i.e. OTUs vs phylotypes) needed before choosing one package to use in analysis. Figure 1 illustrates a decision tree that would be followed in selecting an analysis package.

In our view, the most troublesome aspect of these programs was the use of defaults of which users (or consumers of results) may not be aware. Developers should emphasize default values by presenting a comprehensive table of functions with defaults so users can quickly understand the programs automatic settings and options. All packages are lacking sufficient methods for longitudinal analysis.

Although pipelines aide the researcher in the analysis of microbiome data, the user should always be aware that the purpose of the analysis is not to find the golden P value at the end of program execution. Hypotheses are being tested and the methods used to test these hypotheses make assumptions. The user must be aware of these assumptions and state that study data do or do not meet these assumptions. All scientific studies should be reproducible and therefore users should include enough detail in the methods section of articles so that results can be reproduced.

## Conclusions

Researchers can do well using any one of the seven packages we reviewed here. However, two packages are outstanding; mothur and QIIME, due not only to the comprehensive suite of functions and procedures incorporated into the pipelines but also because of the accompanying documentation. The mothur pipeline offers clear explanations of analysis techniques and provides an easy to follow tutorial. This is an excellent resource for both students and researchers.

## Methods

We included analysis pipelines in this review that met four criteria described below. 1) The software had to be freely available to the general public with no fees associated with its use. 2) The pipeline had to be capable of analyzing data from beginning to end; accepting raw sequencing files directly from the sequencing platform to applying quality control filtering to performing clustering and testing hypotheses. In other words, the pipeline needed to be self contained in that, only one package was required to complete all the tasks necessary in order for a researcher to analysis 16S sequencing data. 3) The pipeline needed to have sufficient public documentation with instructions for downloading and installing the software. Also, the documentation had to be sufficient in that someone who was not formally trained in the use of the pipeline could read the instructions (or tutorials) and successfully upload sequences, read the data, perform quality filtering, run basic analyses/tests, and output results. Troubleshooting resources such as FAQs also needed to be included. 4) The pipeline had to allow for data security. Pipelines that were downloaded directly to a hard drive, had to allow for sequencing data to remain on the hard drive, while web-based pipelines must have included a secure, password protected login system with an option for uploading sequencing data into user-specific secure folders.

After selecting pipelines eligible for review, we researched the documentation to complete Table 1. We also tried each pipeline feature using either the tutorial sequencing data provided or using our own 454 sequencing data. We focused on capabilities and characteristics that would pertain to most researchers.

## Availability of supporting data

**Pipeline Website**

**Mothur**http://www.mothur.org

**Qiime**http://qiime.org

**WATERS**http://code.google.com/p/waters16s

**RDPipeline**https://pyro.cme.msu.edu

**VAMPS**http://vamps.mbl.edu

**Genboree**http://genboree.org

**SnoWMAn**https://snowman.genome.tugraz.at/snowman
